# Reduced Reflex Autonomic Responses Following Intradetrusor OnabotulinumtoxinA Injections: A Pre-/Post-study in Individuals With Cervical and Upper Thoracic Spinal Cord Injury

**DOI:** 10.3389/fphys.2021.796277

**Published:** 2021-12-15

**Authors:** Tristan W. Dorey, Matthias Walter, Andrei V. Krassioukov

**Affiliations:** ^1^Libin Cardiovascular Institute, Department of Cardiac Sciences, Department of Physiology and Pharmacology, Cumming School of Medicine, University of Calgary, Calgary, AB, Canada; ^2^International Collaboration on Repair Discoveries (ICORD), Faculty of Medicine, University of British Columbia, Vancouver, BC, Canada; ^3^Department of Urology, University Hospital Basel, University of Basel, Basel, Switzerland; ^4^Division of Physical Medicine and Rehabilitation, Faculty of Medicine, University of British Columbia, Vancouver, BC, Canada; ^5^G.F. Strong Rehabilitation Centre, Vancouver, BC, Canada

**Keywords:** autonomic dysreflexia, cardiovascular control, heart rate variability, spinal cord injury, urodynamic studies, onabotulinumtoxinA

## Abstract

Urodynamic studies (UDS) can provoke autonomic dysreflexia (AD) in individuals with spinal cord injury (SCI) at and above the sixth thoracic spinal segment potentially leading to profound vagally mediated heart rate (HR) reductions. In this study,^1^ we test the hypothesis that intradetrusor onabotulinumtoxinA injections will improve HR and its variability (HRV) responses to UDS in individuals with cervical and thoracic SCI. A total of 19 participants with chronic SCI (5 women, mean age 42.5 ± 7.9 years) with confirmed neurogenic detrusor overactivity underwent UDS before (i.e., baseline) and 1 month after intradetrusor onabotulinumtoxinA (200 U) injections (post-treatment). Continuous electrocardiography and blood pressure (BP) recordings were used to assess RR-interval, time, and frequency domain metrics of HRV (a surrogate marker of autonomic nervous system activity), and AD pre- and post-treatment. UDS pre-treatment resulted in increased RR-interval as well as time and frequency domain metrics of HRV. Vagally mediated increases in high-frequency (HF) power during UDS were larger in participants with cervical compared to upper thoracic SCI. Post-treatment, UDS had no effect on RR-interval and significantly reduced instances of bradycardia. Furthermore, intradetrusor onabotulinumtoxinA injections significantly reduced time domain metrics of HRV and HF power responses to UDS across all participants. Changes in HRV during UDS could be a potential indicator of improved autonomic cardiovascular function following interventions such as intradetrusor onabotulinumtoxinA injections.

## Introduction

Spinal cord injury (SCI) results in damage to descending autonomic pathways causing a wide array of autonomic dysfunctions (Karlsson, 2006; Krassioukov et al., 2007). Autonomic dysreflexia (AD) is a potentially life-threatening condition characterized by an abrupt increase in systolic blood pressure (SBP) ≥20 mmHg due to innocuous or noxious stimuli below the level of injury (Weaver et al., 2006; Krassioukov et al., 2009). Neurogenic detrusor overactivity (NDO) is the leading cause of AD events in individuals with SCI (Walter et al., 2018a). As such, these individuals require routine urodynamic studies (UDS) for surveillance and management of lower urinary tract (LUT) function (Guttmann and Whitteridge, 1947; Abrams et al., 2008; Groen et al., 2016). AD episodes can be made more serious due to reflexive vagal activation that causes profound bradycardia leading to dysrhythmias, such as atrial fibrillation, sinus pauses, or atrioventricular node block (Pine et al., 1991; Ravensbergen et al., 2012; Manogue et al., 2017).

Considering the high potential risk of cardiovascular complications associated with AD during UDS, as well as in daily living situations (Wan and Krassioukov, 2014), the need for safe and effective treatments that address both NDO and subsequent autonomic consequences is paramount in individuals with SCI. Recently, our group published data from a phase IV clinical trial demonstrating that intradetrusor onabotulinumtoxinA injections are effective at ameliorating AD during UDS while improving LUT function and overall quality of life in individuals with cervical and upper thoracic SCI (Fougere et al., 2016; Walter et al., 2020). Despite this finding, it is still unknown if intradetrusor onabotulinumtoxinA injections can also improve reflex vagal responses to bladder filling during UDS in individuals with SCI.

Heart rate variability (HRV) is a powerful tool used to non-invasively assess autonomic regulation of the cardiovascular system. Reductions in the beat-to-beat variation of RR-interval, the time between two successive R-waves on the electrocardiogram (ECG), are associated with worsened overall health status in a number of disease conditions due to increasing sympathetic tone (La Rovere et al., 2003; Piccirillo et al., 2009; Thayer et al., 2010). In SCI, HRV provides valuable and reliable feedback on the integrity and responsiveness of autonomic pathways in response to external stimuli that is graded by neurological level of injury (NLI; Bunten et al., 1998; Claydon and Krassioukov, 2008; Biering-Sørensen et al., 2018). Specifically, frequency domain analysis of HRV has been shown to be a powerful tool for predicting clinical cardiovascular dysfunction in individuals with SCI (Claydon and Krassioukov, 2008). In the present study, we use HRV to assess autonomic nervous system responses to bladder filling during UDS in individuals with cervical and upper thoracic SCI that underwent the trial. We hypothesized that intradetrusor onabotulinumtoxinA injections would improve reflex autonomic regulation of the cardiovascular system in SCI patients undergoing UDS.

## Materials and Methods

### Study Design and Participants

**Figure 1:**
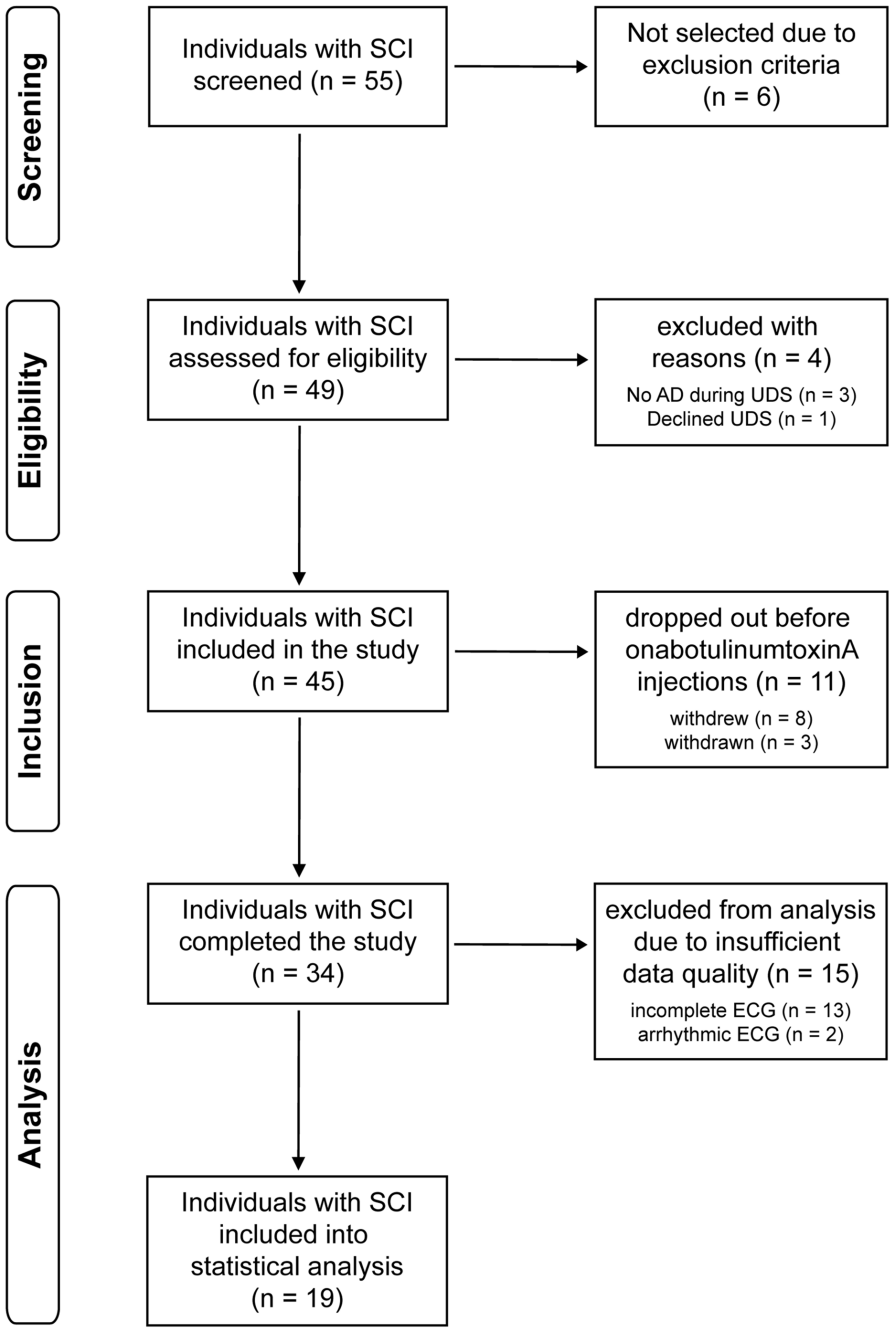
Trial flow diagram. AD, autonomic dysreflexia; ECG, electrocardiogram; SCI, spinal cord injury; UDS, urodynamic study.

This study performed secondary *post hoc* analysis on a cohort from our recent prospective phase IV clinical trial using a pre−/post-study design and was approved by the University of British Columbia Clinical Research Ethics Board (H12-02215) and registered at clinicaltrials.gov (see footnote 1). Between November 2014 and December 2019, 55 individuals with chronic SCI (>1-year post-injury) at or above the sixth thoracic spinal segment (T6) were screened based on inclusion and exclusion criteria reported previously (Fougere et al., 2016). Thirty-four individuals with chronic SCI at T6 or above with confirmed history of AD and NDO were included and assigned to undergo a one-time course of intradetrusor onabotulinumtoxinA injections (200 U) intended to improve LUT function and ameliorate bladder-related AD (Walter et al., 2020). One month following intradetrusor onabotulinumtoxinA injections, UDS were repeated. Of these participants, 13 individuals had incomplete ECG recordings (i.e., not suitable for analysis) and two participants were excluded from analysis due to the presence of arrhythmic events that prevented HRV analysis (Figure 1). In order to perform injury-level-dependent analyses, i.e., cervical versus upper-thoracic SCI, we aimed to match participants with both injury levels by age (within 5 years) for comparison.

### Study Assessments

Neurological level of injury and completeness, i.e., American Spinal Injury Association impairment scale (AIS) grades of SCI, were classified according to the International Standards for Neurological Classification of SCI (Kirshblum et al., 2011). All UDS were performed with a Aquarius TT (Laborie Model 94-R03-BT, Quebec, Canada) in accordance with the International Continence Society’s “Good Urodynamic Practices” (Schäfer et al., 2002). UDS was performed in a supine position and filling speed was standardized across all participants (30 ml/min). Only one UDS was conducted pre- and post-treatment. More information on UDS procedures can be found in the previous clinical trial methods (Fougere et al., 2016). Concurrent to UDS, we continuously recorded beat-by-beat blood pressure, *via* finger photoplethysmography (Finometer PRO, Finapres Medical Systems, Amsterdam, Netherlands) corrected to brachial pressure (CARESCAPE V100, GE Healthcare, WI, United States), and one-lead ECG (eML 132; ADInstruments, CO, United States) for heart rate (HR) in order to detect AD (Walter et al., 2018a,b) and to analyze HRV (see below).

### Heart Rate Variability

Heart rate variability was assessed using time and frequency domain analysis in accordance with the European task force HRV guidelines (Electrophysiology, 1996) and as described elsewhere (Dorey et al., 2019, 2020). Briefly, ECG filtering and R-wave detection was performed using LabChart V8 (ADInstruments, CO, United States). The RR-interval time series were obtained over 4–5-min segments during supine rest and UDS at baseline and post-treatment. Due to the need for at least 4 min of stationary recording for accurate frequency domain analysis UDS measurements were taken from the 4–5 min immediately before the maximum filling volume was achieved. Each segment was manually examined to ensure stationary and stable sinus rhythm with no trend (i.e., average increase or decrease) in RR-interval over the segment. If participants did not have stable sinus rhythm at baseline or during UDS, as classified according to American Heart Association (AHA) guidelines (Kligfield et al., 2007), they were excluded from analysis (Figure 1).

Time domain parameters were calculated for each segment which included the SD of all normal RR-intervals (SDNN) and the root-mean-squared of successive differences in RR-interval (RMSSD). SDNN and RMSSD represent general variability and parasympathetically driven changes in RR-interval, respectively (Berntson et al., 1997). The same segments were subsequently used for frequency domain analysis using Welch’s method with 50% windowing. The periodogram of each segment was then calculated using the Fourier transformation. Total power of each periodogram was measured as a total index of HRV, which is determined by the integral over the entire frequency range. The low-frequency (LF) and high-frequency (HF) components were then extracted. The LF oscillations in HR (0.1–1.5 Hz) are regulated by both the sympathetic and parasympathetic nervous systems, while the HF component (1.5–5 Hz) is predominantly mediated by the phasic activity of the parasympathetic nervous system (Berntson et al., 1997). Spectral analysis is expressed as percent of total power. Beating interval variability was also assessed using nonlinear Poincaré plot analysis. The standard deviations (SD1 and SD2) of each plot were calculated from the RR-interval time series using the following equations:


$$SD1^{2}=2{\left[SD\left(RR_{n}-RR_{n+1}\right)\right]}^{2}\;and\;SD2^{2}={\left[SD\left(RR\right)\right]}^{2}-\frac{1}{2}{\left[SD\left(RR_{n}-RR_{n+1}\right)\right]}^{2}$$

SD1 measures short-term variability and is thus a metric of parasympathetic activity as evident by its correlation with HF power and RMSSD. SD2 is a measure of both short- and long-term variability and correlates with LF-power meaning that it is influenced by both the sympathetic and parasympathetic nervous systems.

### Statistical Analysis

All data are presented as means with SD. Statistical analysis was conducted using Prism version 9.0.0 (GraphPad Software, CA, United States). All data were tested for normality and equal variance by a Kolmogorov–Smirnov test and an F-test, respectively. Data were analyzed using two-way repeated measures ANOVA with Tukey *post hoc* test as indicated in each figure legend. The assumption of sphericity was tested using Mauchly’s test, and the Greenhouse–Geisser correction factor to the degrees of freedom was used for all positive tests. Effect size was calculated using Cohen’s *d*. Correlations between the change in HRV in response to bladder filling during UDS, and SBP changes during UDS and AD were performed using Pearson correlations. A value of *p*<0.05 was considered to be significant.

## Results

In total, 19 individuals (5 women; mean age, 42.5 ± 7.9 years; mean time post-injury, 13.5 ± 11.5 years) were included in the overall analysis (see participant demographics in Supplementary Table S1). To perform injury-level-dependent analyses, all participants in the present cohort with upper thoracic SCI (*n* = 6 mean age, 42.1 ± 8.2 years; mean time-post-injury, 13.5 ± 12.0 years) were assigned age-matched (within 5 years) cervical SCI controls for comparison (*n* = 6 mean age, 43.2 ± 8.0 years; mean time-post-injury, 13.5 ± 11.5 years). Accordingly, no significant difference in age (*p* = 0.462) or time-post-injury (*p* = 0.951) was observed between the two groups. In the whole cohort, majority of participants had motor-complete SCI in accordance with AIS (A = 9, B = 6, C = 3, D = 1).

**Figure 2:**
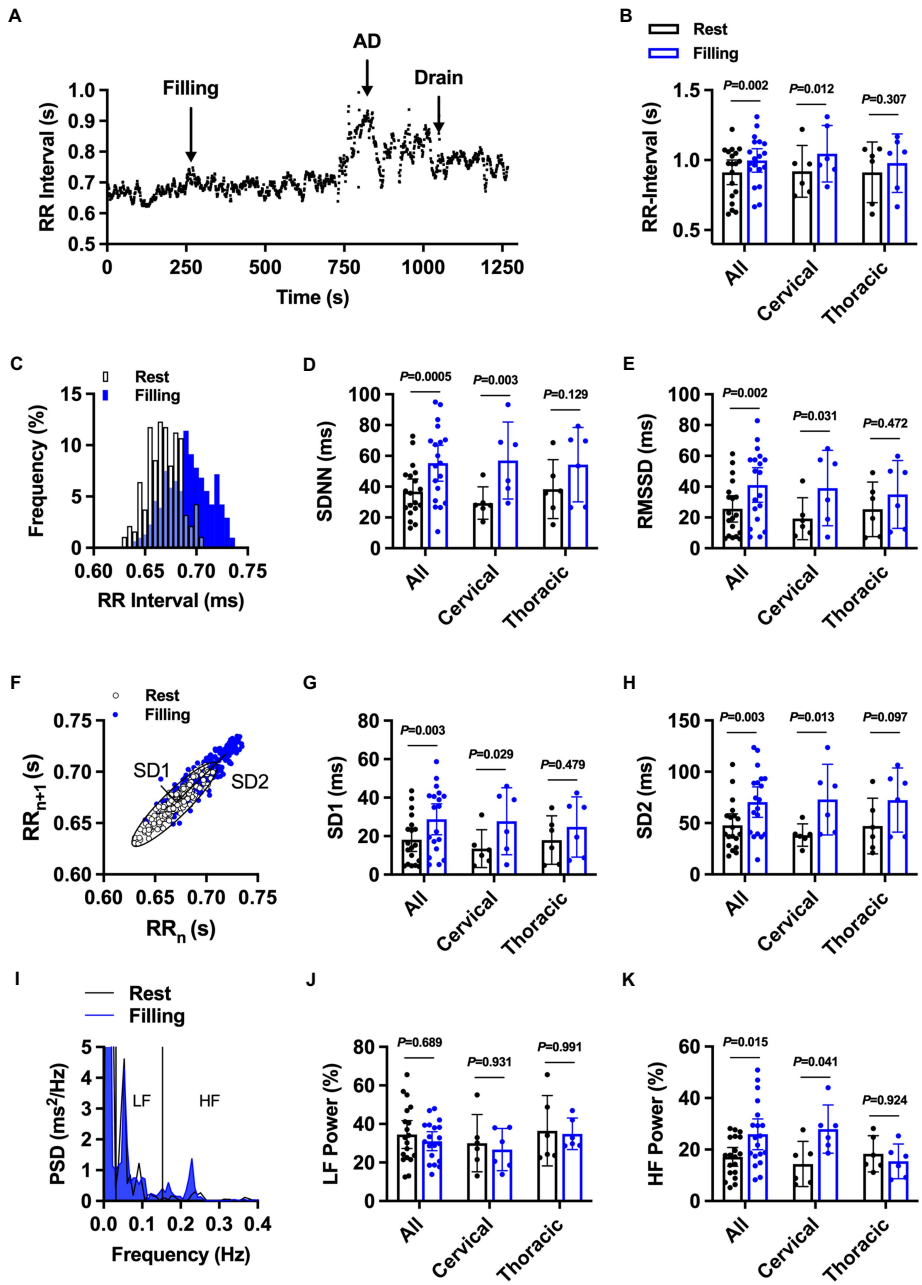
The effect of bladder filling during urodynamic studies on RR-interval and heart rate variability in individuals with cervical (*n* = 13) and upper thoracic (*n* = 6) spinal cord injury. **(A)** Representative RR-interval time series from participant 2 demonstrating prolongation of RR-interval with bladder filling and subsequent bradycardia during autonomic dysreflexia (AD) event. **(B)** Summary RR-interval data at rest and following bladder filling in the whole cohort (all) and in injury-level-dependent subgroups. **(C)** Representative RR-interval histogram at rest and during bladder filling. **(D)** and **(E)**. Summary standard deviation of N-N intervals (SDNN; D) and root mean squared of successive differences (RMSSD; **E**) at rest and during bladder filling. **(F-H)** Representative nonlinear Poincaré plot analysis **(F)** and summary standard deviation 1 (SD1; **G**) and 2 (SD2; **H**) at rest and during bladder filling. **(I-K)** Representative power spectral density plots (I) and summary data for percentage low-frequency (LF; **J**) and high-frequency (HF; **K**) power. Exact values of *p* reported for each comparison *via* two-way repeated measures ANOVA with Holm–Sidak *post hoc* test.

First, we assessed time and frequency domain HRV responses to bladder filling during UDS. Overall, the majority of individuals (57%, 11/19) presented with clinically defined bradycardia during UDS. RR-interval significantly increased in the whole cohort (Figures 2A,B) and in individuals with cervical SCI in response to bladder filling during UDS. There was no change in RR-interval in response to bladder filling during UDS in individuals with thoracic SCI. Bladder filling during UDS increased general variability as assessed by SDNN in the whole cohort (Figures 2C,D) and in cervical SCI but not thoracic SCI. Similarly, parasympathetically driven RMSSD was increased during UDS in the whole cohort (Figure 2E) and in cervical SCI. RMSSD was not significantly different during UDS in thoracic SCI. Nonlinear analysis of HRV assessed by Poincaré plot analysis (Figure 2F) demonstrated significant increases in SD1 (Figure 2G) and SD2 (Figure 2H) in the whole cohort. Cervical SCI also had significant increases in SD1 and SD2. SD1 did not differ in thoracic SCI. While SD2 also did not differ in thoracic SCI during bladder filling, there was a large effect size (Cohen’s *d* = 0.91) following bladder filling. Spectral analysis of LF power was not changed in the whole cohort (Figures 2I,J), cervical SCI, or thoracic SCI in response to bladder filling during UDS. Conversely, HF power increased overall (Figure 2K), in cervical SCI, and did not change in thoracic SCI.

**Figure 3:**
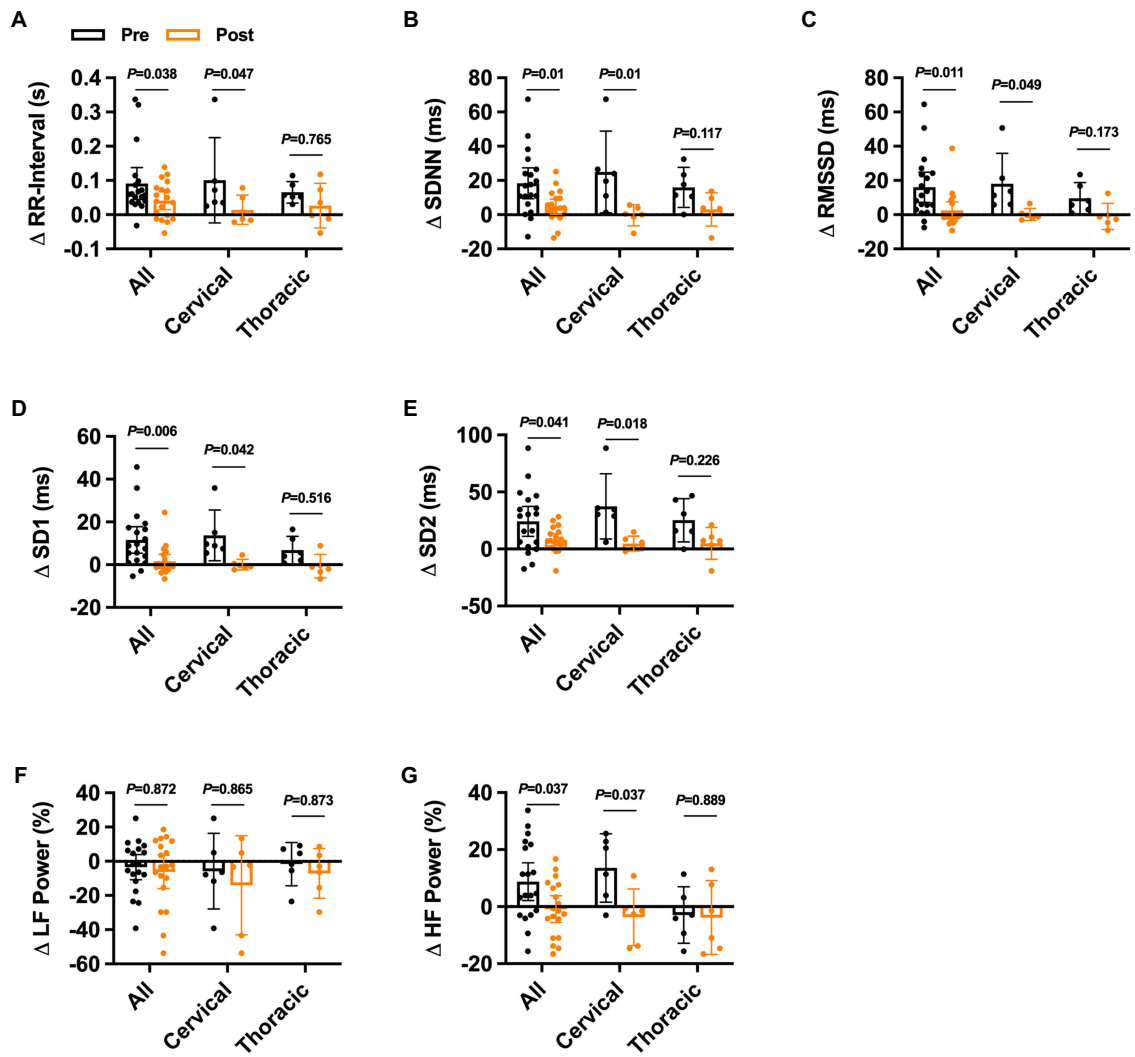
Effects of onabotulinumtoxinA treatment on heart rate variability responses during urodynamic studies in individuals with cervical and upper thoracic spinal cord injury. Pre−/post-onabotulinumtoxinA treatment comparison of the change in **(A)** ΔRR-interval, **(B)** summary standard deviation of RR-intervals (ΔSDNN), **(C)** root mean squared of successive differences (ΔRMSSD), **(D)** standard deviation 1 (ΔSD1), **(E)** standard deviation 2 (ΔSD2), **(F)** percent low-frequency (ΔLF) power, and **(G)** percent high-frequency (ΔHF) power responses to urodynamics UDS in the whole cohort and in injury-level-dependent subgroups. Exact values of *p* reported for each comparison *via* two-way repeated measures ANOVA with Holm–Sidak *post hoc* test.

One-month post-intradetrusor onabotulinumtoxinA injections, cystometric capacity was increased and maximum detrusor pressure was reduced in the whole cohort (Supplementary Table S2). Additionally, the number of participants experiencing NDO and AD during UDS was also reduced. In this sub-cohort, we recorded 6 adverse events {all grade 1 [i.e., fatigue (*n* = 2), pain (*n* = 1); and 2 (i.e., UTI = 3)]} in 5 participants (i.e., 4 with cervical SCI). Furthermore, intradetrusor onabotulinumtoxinA injections reduced the change in RR-interval during UDS in the whole cohort and in cervical SCI (Figure 3A). No differences were observed in the change in RR-interval during UDS in thoracic SCI. Similarly, the change in SDNN was reduced in the whole cohort, in cervical SCI, but not thoracic SCI (Figure 3B). The change in RMSSD, SD1, and SD2 all followed similar trends whereby each was significantly reduced post-treatment in the whole cohort and cervical SCI but was not different in thoracic SCI (Figures 3C–E) Spectral analysis of HRV post-treatment showed no differences in the change in LF power during UDS (Figure 3F); however, HF power (Figure 3G) was reduced in the whole cohort and cervical SCI but unaffected in thoracic SCI.

To assess if the changes in HRV were associated with SBP and UDS parameters through reflex vagal responses, we correlated the change in HRV metrics during UDS for all participants baseline/post-treatment with the changes in SBP during UDS (ΔSBP_UDS_) at the same timepoint (Table 1). Significant correlations were present for ΔRR-interval, ΔRMSSD, ΔSD1, and ΔHF power. We also examined the association between these metrics and the average change in SBP during AD (ΔSBP_AD_). Significant correlations were present for ΔRR-interval, ΔSDNN, ΔRMSSD, ΔSD1, and ΔHF power. Furthermore, ΔRMSSD, ΔSD1, and ΔHF power were significantly associated with maximal SBP during AD.

**Table 1:** Correlations between time and frequency domain responses to UDS and the change in SBP during UDS (ΔSBP_UDS_), AD (ΔSBP_AD_), and maximal SBP during AD.

Category	ΔSBP_UDS_ [mmHg]	ΔSBP_AD_ [mmHg]	Maximum SBP_AD_ [mmHg]
ΔRR-interval [s]	0.421 (0.008)	0.310 (0.05)	0.265 (0.101)
ΔSDNN [ms]	0.218 (0.187)	0.331 (0.042)	0.269 (0.101)
ΔRMSSD [ms]	0.474 (0.002)	0.369 (0.022)	0.328 (0.044)
ΔSD1 [ms]	0.471 (0.003)	0.379 (0.018)	0.332 (0.040)
ΔS2 [ms]	−0.005 (0.975)	0.196 (0.237)	0.145 (0.384)
ΔLF Power [%]	−0.036 (0.827)	−0.068 (0.681)	−0.053 (0.747)
ΔHF Power [%]	0.489 (0.002)	0.428 (0.007)	0.468 (0.003)

*Results are presented as r (Pearson correlation) with p values in brackets. AD, autonomic dysreflexia; HF, high frequency; LF, low frequency; RMSSD, root mean squared of successive differences in RR-interval; SBP, systolic blood pressure; SDNN, SD of RR-intervals; UDS, urodynamic study*.

## Discussion

In the present study, we demonstrate that bladder filling during UDS in individuals with chronic cervical SCI is associated with increased SBP that results in a parasympathetic response characterized by increased RR-interval and HRV. Furthermore, we present evidence that intradetrusor onabotulinumtoxinA injections significantly reduce the HRV responses to bladder filling in this cohort. These findings are in line with the beneficial effects of intradetrusor onabotulinumtoxinA injections that have been reported in the SCI population (Li et al., 2018; Walter et al., 2020).

Regulation of LUT function involves a complex interplay between voluntary motor control and involuntary autonomic control of the central nervous system (Purves et al., 2001; Fowler et al., 2008). In healthy individuals, distention of the urinary bladder results in sympathetic activation and parasympathetic innervation of the detrusor muscles is inhibited (Fowler et al., 2008; Roy and Green, 2019). This prevents involuntary bladder emptying and has shown to result in minor increases in HR and BP as bladder filling increases (Hubeaux et al., 2007). In line with this, Mehnert et al. have shown that in healthy volunteers, HR and LF power both increase throughout bladder filling during UDS, while HF power decreases (Mehnert et al., 2009). However, in SCI, loss of descending motor and autonomic control of the bladder causes NDO that is mediated by spinal reflex pathways (Karlsson, 2006; Fowler et al., 2008). Distention of the urinary bladder sends afferent sympathetic signals that are sustained in spinal reflex pathways during bladder filling which cause vasoconstriction below the level of injury and result in elevated BP and AD (Karlsson, 2006; Fowler et al., 2008). Here, we show that bladder filling during UDS results in a parasympathetic response as seen by increases in RR-interval and well-defined HRV markers of parasympathetic activity such as RMSSD, SD1, and HF power. We hypothesize that this is the result of cardiovagal baroreflex activation secondary to elevated BP during bladder filling. Consistent with this idea, there was a significant association between the change in these markers during bladder filling and the change in SBP at the same timepoint.

Contrary to our findings, two previous studies examined the relationship between UDS and HRV in individuals with SCI and found that HRV did not change in response to bladder filling (Huang et al., 2016; Gomez et al., 2019). Neither study saw any changes in BP during UDS which likely accounts for the lack of an observed effect on HRV. Furthermore, the study by Gomez et al. recruited predominantly individuals with thoracic SCI (Gomez et al., 2019). We observed notable differences in how cervical vs. thoracic SCI participants responded to bladder filling during UDS. Individuals with thoracic SCI did not appear to have any changes in HRV in response to bladder filling during UDS, while cervical SCI participants demonstrated robust increases in RR-interval as well as both time and frequency domain HRV. This is consistent with previous studies showing increased severity of autonomic dysfunction and increased HF power in individuals with cervical SCI compared to thoracic SCI (Claydon and Krassioukov, 2008). These data may suggest that in-tact sympathetic pathways in thoracic SCI are able to effectively oppose reflex vagal activation of the sinoatrial node during UDS.

Injection of onabotulinumtoxinA into the detrusor results in block of the pre-synaptic release of acetylcholine from the parasympathetic innervation to produce partial paralysis of the detrusor muscle (Fowler et al., 2008). This in turn results in minimized reflexive sympathetic activation of spinal reflex pathways and lowers SBP responses to bladder filling (Walter et al., 2020). Consequently, lower SBP responses to bladder filling cause diminished activation of the cardiovagal baroreflex (Claydon and Krassioukov, 2008). Consistent with our findings demonstrating that treatment had no effect on HRV at rest, Mehnert et al. have published similar findings showing intradetrusor onabotulinumtoxinA injections had no impact on time or frequency domain parameters in participants with NDO (Mehnert et al., 2016). Despite this, their study did not examine how HRV changed under physical stress, such as UDS. Accordingly, in the present study, HRV analysis post-treatment revealed reduced reflex vagal responses to bladder filling as evident by reductions in the change of HF power during UDS. We also found a significant association between the change in markers of parasympathetic activity (RMSSD, SD1, and HF power) during bladder filling and the severity of AD. These data may provide some preliminary evidence for the use of HRV monitoring during UDS in order to predict AD events. However, because this study is a secondary analysis, we acknowledge that the sample size may not be adequately powered to determine the sensitivity and specificity of HRV as a predictor of AD.

This study highlights the potential for intradetrusor onabotulinumtoxinA injections to minimize reflex autonomic responses associated with UDS in this cohort. Our study does, however, have some limitations. It is well known that respiratory rate can have a significant effect on HRV and was not controlled in the present study. However, the robust and significant effects of our intervention suggest that the impacts of UDS on HRV are larger than those imposed by alterations in respiratory rate. Additionally, a larger sample size including a wider array of injury levels would improve our studies generalizability and improve the use of HRV as a prognostic tool. It will also be important for future studies to assess how both resting HRV and changes in HRV in response to physiological stimuli such as NDO relate to long-term cardiovascular risk in this population.

## Conclusion

The findings in the present study show that UDS increases reflex parasympathetic activation, as assessed by HRV, in response to bladder filling during UDS and that intradetrusor onabotulinumtoxinA injections ameliorate this response in individuals with SCI. These findings align with previous studies highlighting the beneficial effects of intradetrusor onabotulinumtoxinA injections in individuals with SCI. Furthermore, we provide evidence that HRV is correlated with both AD and UDS metrics. Thus, it may prove to be a useful monitoring tool for early prediction of AD during UDS.

## Data Availability Statement

The raw data supporting the conclusions of this article will be made available by the authors, without undue reservation.

## Ethics Statement

The studies involving human participants were reviewed and approved by University of British Columbia Clinical Research Ethics Board. The patients/participants provided their written informed consent to participate in this study.

## Author Contributions

AK and MW had full access to all the data in the study and take responsibility for the integrity of the data and the accuracy of the data analysis. TD, MW, and AK contributed to conception and experimental design and interpreted the data. MW and AK acquired the data and supervised the project. TD analyzed the data and drafted the manuscript. All authors contributed to editing the final version of the manuscript.

## Funding

TD (Canadian Institutes for Health Research Doctoral Research Award, number 412708), MW (Postdoctoral Research Trainee Award from the Michael Smith Foundation for Health Research in partnership with the Rick Hansen Foundation under Grant number 17110 and Vancouver Coastal Health Research Institute Rising Start Award), AK (Praxis Spinal Cord Institute, i.e., formerly Rick Hansen Institute, Grant number G2013-09 and Allergan Inc. for providing study drug Botox in-kind). AK also holds the Endowed Chair in Rehabilitation Medicine.

## Conflict of Interest

The authors declare that the research was conducted in the absence of any commercial or financial relationships, that could be construed as a potential conflict of interest.

## Publisher’s Note

All claims expressed in this article are solely those of the authors and do not necessarily represent those of their affiliated organizations, or those of the publisher, the editors and the reviewers. Any product that may be evaluated in this article, or claim that may be made by its manufacturer, is not guaranteed or endorsed by the publisher.
