# Supplementary material for: Reduced Reflex Autonomic Responses Following Intradetrusor OnabotulinumtoxinA Injections: A Pre-/Post-study in Individuals With Cervical and Upper Thoracic Spinal Cord Injury
Source: Front Physiol. 2021 Dec 15;12:796277. doi: 10.3389/fphys.2021.796277 (PMC8769099; doi:10.3389/fphys.2021.796277)
Supplement: Supplementary file 2 [file Table_1.pdf]

# *Supplementary Material – Dorey, Walter and Krassioukov*

*doi:10.3389/fphys.2021.796277*

**Supplementary Table 1 – Participant and injury characteristics.**

| **No.** | **NLI** | **AIS** | **Age* (years)** | **Sex** | **TPI* (years)** | **History of OnabotulinumtoxinA injections** |
|---|---|---|---|---|---|---|
| 1 | C1 | C | 41-45 | M | 26-30 | Naive |
| 2 | C4 | A | 31-35 | F | 11-15 | Naive |
| 3 | C4 | D | 41-45 | M | 1-5 | Naive |
| 4 | C5 | B | 31-35 | M | 1-5 | Naive |
| 5 | C5 | C | 61-65 | M | 1-5 | Naive |
| 6 | C5 | C | 41-45 | M | 21-25 | Naive |
| 7 | C5 | B | 31-35 | M | 1-5 | Yes |
| 8 | C6 | A | 41-45 | F | 11-15 | Naive |
| 9 | C7 | A | 41-45 | F | 16-20 | Naive |
| 10 | C7 | B | 36-40 | M | 1-5 | Naive |
| 11 | C7 | B | 46-50 | M | 6-10 | Naive |
| 12 | C8 | A | 41-45 | M | 21-25 | Naive |
| 13 | C8 | B | 41-45 | M | 36-40 | Naive |

| | | | | | | |
|---|---|---|---|---|---|---|
| 14 | T2 | A | 36-40 | M | 6-10 | Yes |
| 15 | T3 | B | 36-40 | M | 16-20 | Yes |
| 16 | T4 | A | 36-40 | F | 21-25 | Naive |
| 17 | T4 | A | 56-60 | M | 1-5 | Naive |
| 18 | T5 | A | 41-45 | F | 26-30 | Yes |
| 19 | T5 | A | 46-50 | M | 1-5 | Naive |

* For information, such as age and time post injury, that would allow the study participant or their family, friends or neighbors to identify them, we chose to provide a 5-year range for age and time post injury, rather than revealing the actually numbers.

AIS; American spinal injury impairment scale, C; cervical, F: female, M: male, NLI; neurological level of injury, SD; standard deviation, T; thoracic, TPI; time post-injury, yr: years
