# Supplementary material for: Reduced Reflex Autonomic Responses Following Intradetrusor OnabotulinumtoxinA Injections: A Pre-/Post-study in Individuals With Cervical and Upper Thoracic Spinal Cord Injury
Source: Front Physiol. 2021 Dec 15;12:796277. doi: 10.3389/fphys.2021.796277 (PMC8769099; doi:10.3389/fphys.2021.796277)
Supplement: Supplementary file 3 [file Table_2.pdf]

Supplementary Material – Dorey, Walter and Krassioukov

doi:10.3389/fphys.2021.796277

Supplementary Table 2 – Post-treatment changes of urodynamic parameters and systolic blood pressure across all participants.

	**All [n=19]**		
	**Pre**	**Post**	***p* value**
**Urodynamic parameters**			
Cystometric capacity [mL]	377 [200 -518]	600 [477-720]	<0.001
Pdetmax [cmH_2O]	41 [30 - 53]	14 [9-22]	<0.001
Presence of NDO [n]	19	12	0.008
**SBP changes during UDS**			
Baseline SBP [mmHg]	108 [103-118]	113 [104-122]	0.9
Maximum SBP [mmHg]	160 [142-171]	140 [120-166]	0.02
Δ SBP [mmHg]	48 [30-60]	23 [18-55]	0.002
Presence of AD [n]	19	12	0.008

Data is presented as medians with interquartile ranges [25% - 75% IQR]. Wilcoxon signed-rank test was used for all pre-/post-treatment comparison, except for presence of NDO and AD (i.e. Fisher's exact test).

AD = autonomic dysreflexia, NDO = neurogenic detrusor overactivity, Pdetmax = maximum detrusor pressure, SBP = systolic blood pressure, UDS = urodynamic studies.
